# Analysis of Antimicrobial Use and the Presence of Antimicrobial-Resistant Bacteria on Austrian Dairy Farms—A Pilot Study

**DOI:** 10.3390/antibiotics11020124

**Published:** 2022-01-18

**Authors:** Clair L. Firth, Annemarie Käsbohrer, Peter Pless, Sandra Koeberl-Jelovcan, Walter Obritzhauser

**Affiliations:** 1 Unit of Veterinary Public Health and Epidemiology, Institute of Food Safety, Food Technology & Veterinary Public Health, University of Veterinary Medicine, 1210 Vienna, Austria; 2 Veterinary Directorate and Administration, Styrian Provincial Government, 8010 Graz, Austria; 3 Institute for Medical Microbiology and Hygiene, Centre for Foodborne Infectious Diseases, Division for Public Health, Austrian Agency for Health and Food Safety (AGES GmbH), 8010 Graz, Austria; 4 Veterinary Practice, 8605 Parschlug, Austria

**Keywords:** antimicrobial resistance, antibiotics, dairy, ESBL, MRSA, farms, veterinary

## Abstract

The assumed link between high levels of antimicrobial use on farms and selection for antimicrobial-resistant (AMR) bacteria on that farm remains difficult to prove. In the pilot study presented here, we analysed total antimicrobial use on 50 dairy farms in Austria and also collected environmental samples to ascertain whether specific AMR bacteria were present. Antimicrobial use (AMU) analysis was based on electronic veterinary treatment records over a one-year period. Faecal samples for the assessment of extended-spectrum beta-lactamase (ESBL)-producing *E. coli* were collected from cowsheds, calf pens, and youngstock housing areas, as well as dust samples from barns, to isolate methicillin-resistant *Staphylococcus aureus* (MRSA). Bacteriological cultures were carried out on selective agar. Farms were split into groups of 25 of the highest antimicrobial users and 25 of the lowest users. Overall, samples from 13/50 (26.0%) farms were found to be positive for the presence of ESBL-producing *E. coli*. Of these, eight farms were in the low user group and five were in the high user group. Only one farm was confirmed to harbour MRSA. Statistical analyses demonstrated that there was no significant difference in this study population between high or low antimicrobial use with respect to the presence of ESBL-producing *E. coli* on farms (*p* = 0.33). In conclusion, the presence of specific AMR bacteria on farms in this study population was not found to have a statistically proven relationship with their level of antimicrobial use.

## 1. Introduction

Globally, the excessive use of antimicrobials and the increasing level of antimicrobial-resistant bacterial infections in both humans and animals are continuing to cause concern among veterinarians, medics, and the general public [1]. Veterinary antimicrobial use worldwide is expected to increase by an estimated 11.5% by 2030 (based on 2017 figures), and so the problem will persist [2]. While many countries (such as China, Brazil, the USA, and Australia) continue to misuse antimicrobials for non-therapeutic growth promotion, such use has been banned in the European Union (EU) since 2006 [3,4]. In Austria, antimicrobials for use in animals are only available from veterinarians and never over the counter. Injectable antimicrobials are further restricted and can only be dispensed to farmers who are members of the Animal Health Service (*Tiergesundheitsdienst*) and have completed a specific training course in medication administration and documentation. Since 2015, all antimicrobials dispensed for use in food-producing animals by veterinarians must also be reported to the relevant authorities [5]. Based on the latest national reports (for 2020), the vast majority of dispensed antimicrobials (73.4%) are provided for use in Austrian pig production, while 19.7% are for use in cattle (of which, around 7% are for dairy cows and 9% for beef cattle) and the remaining 6.7% for use in poultry [6].

Whilst a proven relationship between antimicrobial use (AMU) and the selection for antimicrobial-resistant (AMR) bacteria remains a contentious issue, a number of studies have assessed the possibility of such a correlation. In 2014, a report on seven European countries (including Austria) determined a strong association between total antimicrobial use in livestock in each country and the reported prevalence of AMR in *E. coli* in those countries [7]. Similarly, a study of outpatient antimicrobial consumption and AMR in human patients determined a strong linear relationship between macrolide use and resistant *S. pneumoniae* (MRSP) in 16 countries (including Austria) [8]. Meanwhile, a study from the Netherlands determined no association between total AMU on farms and the presence of ESBL/AmpC-producing *E. coli*, although a correlation between increasing third- and fourth-generation cephalosporin use and increasing levels of these cephalosporin-resistant bacteria was detected [9]. More recently, a Swedish study of antimicrobial use on dairy farms analysed the presence of resistant phenotypes among *E. coli* and found no link between overall AMU and *E. coli* resistance [10]. However, the study’s authors noted that AMU in Sweden is generally much lower than in other EU countries [10].

With respect to the risk to humans from antimicrobial-resistant bacteria in food, a number of studies have found evidence of AMR bacteria [11,12,13], including an Austrian study in 2014 which reported the presence of ESBL-producing *E. coli* in 20% of minced pork/beef samples tested, as well as MRSA in 9% of these samples [14]. As part of the EU AMR monitoring system, the European Food Standards Agency (EFSA) and the European Centre for Disease Control and Prevention (ECDC) publish joint annual summary reports on AMR in zoonotic and indicator bacteria from humans, animals, and food. The Austrian health authorities provide data for these reports and the most recent national monitoring results in 2019 reported the isolation of ESBL/AmpC-producing *E. coli* in 4 of 340 (1.2%) beef samples, as well as MRSA in 6 of 228 (2.8%) beef samples [15,16,17]. A recent pan-European study of 11 countries, including Austria, failed to detect a statistically significant association between ESBL and/or AmpC-producing *E. coli* (i.e., those resistant to third-generation cephalosporins) in humans and calves under one year of age [18]. Nevertheless, a source attribution model of ESBL-producing *E. coli* in the Netherlands highlighted that transmission to and from non-human sources (including cattle) is necessary to continue the intra-community spread of ESBL-producing *E. coli* and plasmid-mediated AmpC-producing *E. coli* [19].

The study presented here represents initial data from a pilot study of a small group of dairy farms, where extensive data on antimicrobial use over a one-year period, as well as farm management practices, were available. These data, as well as whether farms were considered “high” or “low” users of antimicrobials relative to the study population, were compared with the prevalence of ESBL-producing *E. coli* and MRSA obtained from samples collected on farms.

## 2. Results

In total, 138 voided faecal samples were collected from 50 farms (25 classified as “high” antimicrobial users and 25 as “low” users). Samples from the areas of the barn where cows were kept were available from all 50 farms. Calves were present on 46/50 farms (92.0%) and pooled samples (1–5 animals per pool) were taken from calf holding areas/hutches. Youngstock (>six months of age) were present on 32/50 farms (64.0%) and pooled samples (1–5 animals per pool) were taken from their pens. An additional ten pooled samples were taken from calves and youngstock on four farms, which reared more than 50 head of cattle. Dust samples were taken from all 50 farms.

### 2.1. Farm Population

In the “high” AMU group, the mean herd size was 47.3 head of cattle (median 38; range 14–128), including 22.6 dairy cows (median 17; range 8–56); whereas in the “low” AMU group, the mean herd size was 57.1 head of cattle (median 52; range 11–157), including 29.6 dairy cows (median 24; range 5–77). In the high use group, 17/25 (68.0%) farms were run conventionally, while just 2/25 (8.0%) farms were organic producers. No production type was reported for the remaining six farms (24.0%) as the farmers did not complete the farm management survey (as previously described elsewhere [20]). In the low use group, 7/25 (28.0%) farms were run organically, 14/25 (56.0%) conventionally, and production type was not known for the remaining 4 farms. Of the 40 farmers in the overall study group who completed the farm management survey, 27 (67.5%) reported that they routinely fed waste/discard milk containing antimicrobial residues (from the treatment of cows) to calves on their farms. For further details, see Table 1.

### 2.2. Bacteriology of Farm Samples

ESBL-producing *E. coli* were isolated in faecal samples from 13 (26%) of the 50 participating farms. Of these 11 pooled calf samples, seven pooled cowshed boot swab samples and four pooled youngstock samples were positive (for details, see Table 1). Three “high use” farms were found to be positive in all types of bovine faecal samples (i.e., cows, calves, and youngstock); while two of the “low use” farms were positive in both the cow and calf sample types and no youngstock were present at the time of sampling. One “high use” farm kept enough calves and youngstock (>5 head in each group) to require two pooled samples to be taken; however, these additional samples were negative, as were the other samples from this farm. In total, additional pooled samples were taken from three “low use” farms with sufficient animals. On two of these farms, all samples were negative and on one farm, the samples from cows and calves were positive for ESBL-producing *E. coli* in both the standard and additional samples.

Dust samples from only one farm were found to be positive for MRSA, this isolate was found to contain the *mecC* gene.

### 2.3. Antimicrobial Use Data

The total Defined Daily Doses per cow and year (DDDvet/cow/year) for all disease indications on each farm was calculated according to the standardised Defined Daily Dose (DDDvet) values published by the European Medicines Agency [21]. Total antimicrobial use (AMU) for all disease indications ranged from 2.47 to 8.04 DDDvet/cow/year in the high use group (mean 4.35; median 3.82 DDDvet/cow/year), compared to 0.01 to 0.63 DDDvet/cow/year in the low use group (mean 0.29; median 0.31 DDDvet/cow/year). As would be expected, the two groups (“high” and “low” AMU in DDDvet/cow/year) of antimicrobial use showed a highly statistically significant difference in the Mann–Whitney U-test (U score 0, Z score 4.88, *p* < 0.00001).

Among high antimicrobial users on ESBL-producing *E. coli* positive farms, the median AMU was 3.38 DDDvet/cow/year (mean 3.77, *n* = 5); compared to the negative farms where the median AMU was 4.14 DDDvet/cow/year (mean 4.50, *n* = 20). On farms classed as low users, the median AMU was 0.40 DDDvet/cow/year (mean 0.31, *n* = 8) on farms where at least one sample tested positive, compared to a median of 0.29 DDDvet/cow/year (mean 0.28, *n* = 17) on negative farms.

**Figure 1:**
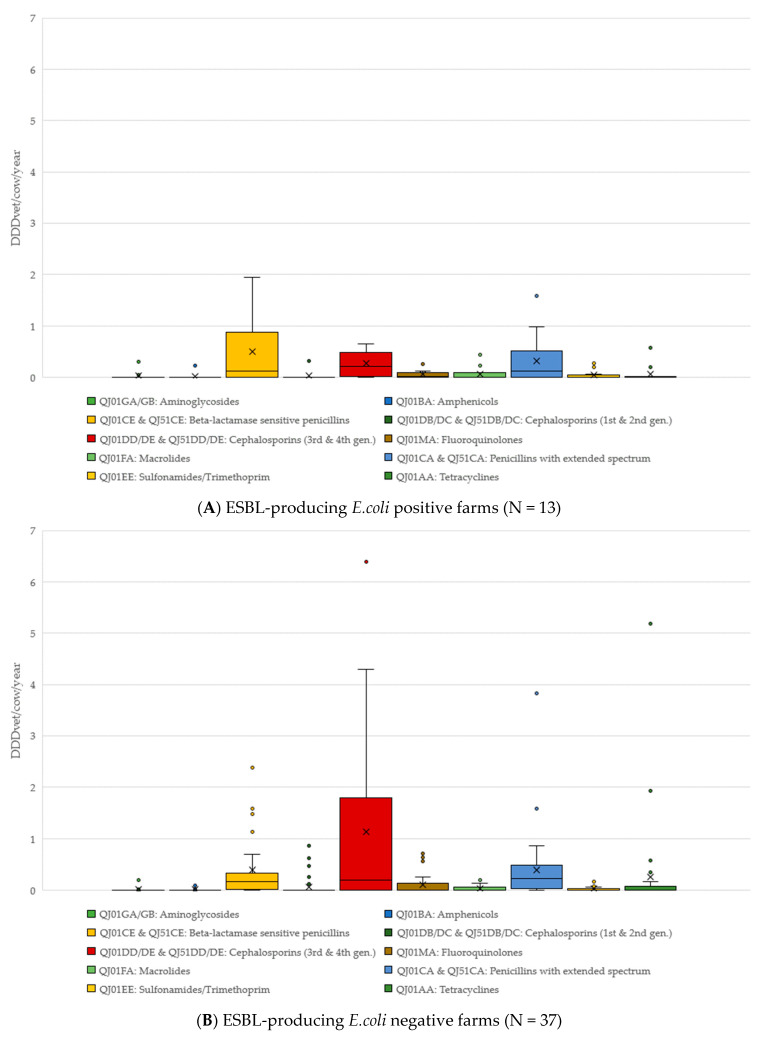
Antimicrobial treatments by antimicrobial class (and ATCVet code), divided into those farms where faecal samples tested positive for ESBL-producing *E. coli* (**A**) and those where samples tested negative for ESBL-producing *E. coli* (**B**).

**Figure 2:**
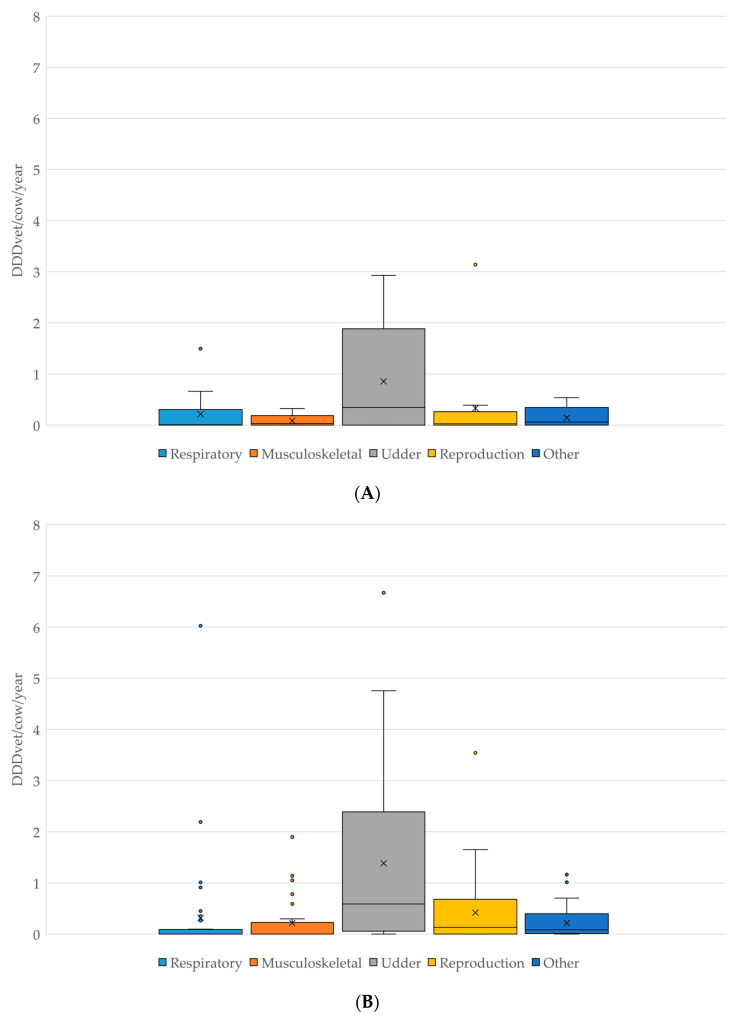
(**A**): Comparison of antimicrobial use in DDDvet/cow/year by disease indication on farms which tested POSITIVE for the presence of ESBL-producing *E. coli* (N = 13). (**B**): Comparison of antimicrobial use in DDDvet/cow/year by disease indication on farms which tested NEGATIVE for the presence of ESBL-producing *E. coli* (N = 37). X—mean; horizontal line—median; box—range between 1st and 3rd quartile; dots—outliers.

The comparison of antimicrobial classes used on both ESBL-producing *E. coli* positive and negative farms is shown in Figure 1. On ESBL-producing *E. coli* positive farms, beta-lactamase sensitive penicillins (such as benzylpenicillin) had the highest DDDvet/cow/year (mean 0.49, median 0.12, maximum 1.95 DDD/cow/year); while the DDDvet/cow/year for third/fourth-generation cephalosporins was higher on the ESBL-producing *E. coli*-negative farms (mean 1.13, median 0.19, maximum 6.39 DDD/cow/year) (for details, see Figure 1). As would be expected on dairy farms, the majority of antimicrobial treatment on all farms was for udder diseases (53.8% as calculated as a total of the DDDvet/cow/year), followed by a much lower proportion of treatments for reproductive disorders (17.8% of the total DDDvet/cow/year) (for details, see Table 2 and Figure 2).

**Figure 3:**
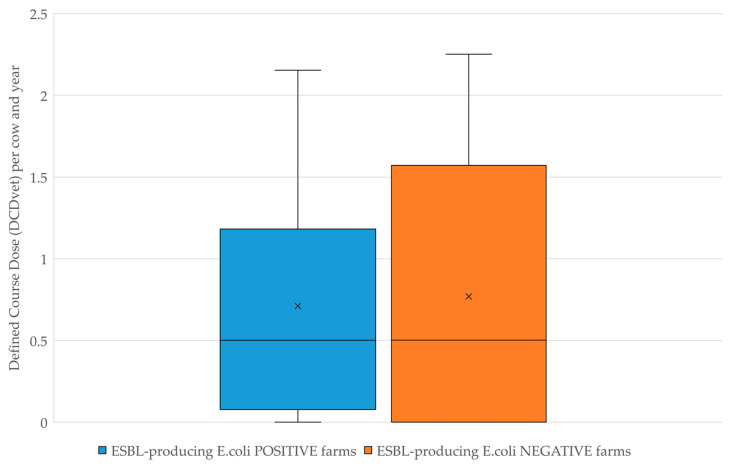
Comparison of antimicrobial dry cow therapy in Defined Course Dose per cow and year (DCDvet/cow/year).

Dry cow therapy was analysed by Defined Course Dose (DCDvet) per cow and year and is shown in Figure 3. Among farms where at least one sample tested positive for the presence of ESBL-producing *E. coli*, the median was 0.53 DCDvet/cow/year (mean 0.71), whereas on negative farms the median was 0.50 DCDvet/cow/year (mean 0.77).

### 2.4. Statistical Analysis

Using the Mann–Whitney U-test for independent samples, no statistically significant difference was determined between AMU in DDDvet/cow/year on each of the farms which were classified as ESBL-producing *E. coli* positive or negative (Mann–Whitney U score 187.0, Z score 1.17, *p* = 0.24). With respect to the use of third and fourth generation cephalosporins, the Mann–Whitney U-test similarly showed no statistically significant difference between the levels of use of this antimicrobial group regardless of whether ESBL-producing *E. coli* were isolated on the farm or not (Mann–Whitney U score 206.5, Z score 0.74, *p* = 0.46).

Using the Chi-squared test, no statistical significance was found at the 5% level between ESBL-positive *E. coli* or negative farms with respect to either “high” (≥2.47 DDDvet/cow/year) or “low” (≤0.63 DDDvet/cow/year) use groups (*p* = 0.33), or the number of dairy cows (*p* = 0.42) (Table 3 and Table 4).

With respect to feeding calves waste milk, no statistically significant difference was determined for the presence of ESBL-producing *E. coli* between those farms routinely feeding calves waste milk containing antimicrobial residues and those which did not (Fisher’s exact test, *p* = 0.44) (Table 5).

## 3. Discussion

To the authors’ knowledge, this is the first study to analyse the presence of antimicrobial-resistant bacteria in relation to antimicrobial use on Austrian dairy farms. The current study represents the first steps in data collection on 50 dairy farms participating in a larger study of 250 farms. Analyses of faecal samples from the farm environment (boot swabs from alleyways of the cowshed, pooled faecal samples from calves and youngstock) determined a low prevalence of ESBL-producing *E. coli*, with approximately one quarter (26.0%) of the 50 farms testing positive in at least one sample. This is relatively low in comparison to neighbouring Bavaria (Germany) where a previous study of dairy and beef farms reported at least one positive ESBL-producing *E. coli* sample was found on 86.7% of the 45 farms tested [23]. Similarly, a study in the Netherlands collected ESBL/AmpC-positive samples on 41% of 100 conventionally run dairy farms [9]. A follow-up to this Dutch study carried out two years later after a change in local legislation to restrict the use of third- and fourth-generation cephalosporins demonstrated that the herd prevalence between matching herds fell from 32.7% ESBL/AmpC *E. coli*-positive in 2011 to 18.0% in 2013 [24]. However, the results determined here in Austria are not surprising as they correspond to the most recent official statistics published by the European Food Safety Authority and the European Centre for Disease Prevention and Control in 2020, where 20.5% of slaughtered calves (<1 year of age) in Austria were found to have presumptive ESBL-producing *E. coli* in their caeca, compared to a similar prevalence in Switzerland (19.1%), slightly higher in the Netherlands (36.4%), and much higher prevalences in Germany (66.8%), Belgium (64.8%), and Italy (86.8%) [25].

The statistical analysis presented here demonstrated that there was no statistically significant correlation between farms classified as “high” (≥2.47 DDDvet/cow/year) antimicrobial users or “low” (≤0.63 DDDvet/cow/year) antimicrobial users in this study population and the presence of ESBL-producing *E. coli* on these farms. This trend has also been reported in the Netherlands, where researchers found that the total annual animal-defined daily dose (DDDA) did not significantly differ between ESBL/AmpC-positive and negative farms [9,26]. Furthermore, the results presented here did not show a relationship between the presence of ESBL-producing *E. coli* and the use of third and fourth generation cephalosporins, contrasting with a Dutch study which found the use of these highest priority critically important antimicrobials (HPCIAs) led to an 8.05-fold increase in the odds of testing positive for ESBL/AmpC-producing *E. coli* [9]. A German study comparing dairy farms, which either did or did not use antimicrobials for a one-year period prior to sampling, found that 30% of the 10 control farms (no antimicrobial use) were ESBL-producing *E. coli* positive, while 39/45 (86.7%) of the dairy farms using antimicrobials tested positive [23]. A recent pan-European analysis on the prevalence of ESBL and/or AmpC-producing *E. coli* in slaughtered veal calves aged under one year (as well as broilers, turkeys, and fattening pigs) has also shown a statistically significant association between the national consumption of third and fourth generation cephalosporins in food-producing animals and the presence of these AMR bacteria (logistic regression based on national data from 31 countries, including Austria, in 2017–2018, odds ratio 1.29 (95% CI: 1.14; 1.46), *p* < 0.001) [18].

A study in France demonstrated that young calves (<7 weeks of age) on dairy farms were harbouring a variety of AMR bacteria in their intestinal microbiome [27]. In particular, this study demonstrated that the proportion of ESBL-producing *E. coli* as determined by selective media, fell slightly from 22% at 15 days of age to 19% at 7 weeks [27]; a trend which has been observed in many other studies of the intestinal flora of dairy calves [28,29,30,31]. While the feeding of waste milk containing antimicrobial residues to calves did not appear to have a statistically significant effect on the farms sampled in the current study, a number of other studies have shown that feeding such milk to calves leads to a transient increase in the presence of AMR bacteria in their faeces [31,32,33,34]. It is important to note that, similar to a Canadian study investigating extended-spectrum cephalosporin-resistant *E. coli* in calf faeces [34], the survey of farm management practices in the current study only provided “herd level” information and did not confirm that the calves sampled here had actually received waste milk containing antimicrobial residues, only that it was a routine practice on that farm. Furthermore, we did not investigate the impact of the prevalence of AMR bacteria on this practice.

Although data on MRSA on dairy farms are limited, other European studies in dairy cattle have reported a much higher prevalence of MRSA than that determined in the present study, where only one farm tested positive. A German study of three dairy herds more than a decade ago found that 46.7% (7/15) of cows tested were MRSA-positive and at a similar time, a Belgian study reported 9.3% of the 118 dairy farms tested were MRSA-positive [35,36]. The results determined for MRSA on these 50 Austrian farms are, however, comparable to the extremely low proportion of cattle testing positive for MRSA in a study of patients at the University of Veterinary Medicine in Vienna’s ruminant clinic, where only 0.45% (95% CI: 0.01; 2.90%) of 221 cattle were MRSA-positive [37].

A previous study of human patients in the federal state of Upper Austria reported that 16/21 (76.2%) patients testing positive for MRSA CC398 were either pig or poultry farmers or had relatives with direct animal contact [38]. The Austrian researchers authoring that study suggested that the high level of doxycycline resistance (19/21, 90.5%) of MRSA isolates determined among these MRSA-positive patients could be connected to the relatively high proportion of tetracycline use on pig and poultry farms in Austria at this time [38]. However, a more recent Austrian study reported that human patients were much more likely to become infected with resistant *S. aureus* just through living in rural areas rather than working directly in animal production (OR 1.53 (95% CI: 1.02; 2.30) vs. OR 0.54 (95% CI: 0.19; 1.55)), although this may have been due to the relatively small number of livestock farmers (96/3309; 2.9%) included in that study population [39].

A study from the United States reported that, of 18 dairy farms sampled, 50% were positive for antimicrobial-resistant *Enterobacteriaceae* in environmental sampling and that on three (17%) of these farms such strains were isolated from 75.1 to 100% of all tested surfaces [40]. This study found that over 60% of “shared human and animal contact surfaces” were positive for AmpC and over 20% were positive for ESBL-producing *Enterobacteriaceae*, a finding which is extremely relevant for the possible transfer of antimicrobial resistance from animals to humans [40].

A meta-analysis of 181 studies to assess possible associations between antimicrobial use in food-producing animals and antimicrobial resistance in such animals and humans was published in 2017 [41]. A total of 81 studies on animals and 13 studies on humans were included in the final meta-analysis. In this study, the pooled absolute risk differences when interventions were introduced into livestock farming systems to reduce antimicrobial use led to a 10 to 15% (range 1–39%) lower proportion of resistant isolates in these systems compared to the control group, where no interventions were implemented [41]. The authors of this meta-analysis reported that there is sufficient evidence to demonstrate that a transfer of AMR bacteria can occur between livestock and farm workers and that this appears to occur less frequently when AMU in animal populations is reduced. However, they also pointed out that farmworkers are also the most commonly investigated group and that the evidence is, therefore, weaker for the general human population [41].

While the milk produced in this study population was not specifically tested for ESBL-producing *E. coli*, other European studies have investigated the presence of these AMR bacteria in the milk. A German study of bulk tank milk detected ESBL-producing *Enterobacteriaceae* in 9.5% of the 866 dairy farms tested [42], while a Swiss study of 100 dairy farms did not determine any ESBL-producing *E. coli* [43] in saleable milk.

In the present study, no statistically significant relationship could be proven between AMU and AMR on farms. It is important to note, however, that AMU on these Austrian dairy farms was relatively low compared to the dairy industry in other European countries [44,45,46,47], as well as the pig and poultry sector as a whole, and it is also unlikely that definitive conclusions can be made based on the small number of samples isolated which tested positive for ESBL-producing *E. coli*.

The main limitation of the current study was that the study population was not randomised and the data on antimicrobial use in this population of dairy cows cannot, therefore, be extrapolated to the rest of the Austrian dairy cow population. However, given the potentially sensitive nature of collecting diagnosis and antimicrobial treatment data directly from herd veterinarians, as well as the high level of commitment required by both veterinarian and farmer to complete all required study tasks, the decision was made to invite veterinarians to participate (convenience sampling) and that they should, in turn, suggest farmers from their client base to join the study (respondent-driven sampling). The subpopulation of 50 farms included here to investigate AMR bacteria was actually part of a much larger study containing around 250 dairy farms. Over a one-year period, the following data were collated from the 250 farms: AMU, bacteriological milk culturing results, veterinary diagnoses, milk recording results, animal movement data, and many other farm management factors [20,44]. While it is true that the enrolment of the 50 farms in the current study does indeed introduce a certain level of bias into the study population, we are of the opinion that this bias was limited as neither the farmers nor their herd vets knew which farms would be sampled for AMR bacteria at enrolment. The larger observational study commenced in October 2015, the faecal/dust samples included here were taken in July 2017, and the farms included in the current study were based on their AMU in 2015–2016. We do not believe that the herd veterinarians were able to influence the inclusion or exclusion of specific farms with antimicrobial resistance problems as the initial enrolment of farmers took place in winter 2014/2015, i.e., more than two years prior to AMR samples being collected.

A further limitation may have been the decision to include only the highest and lowest AMU farms in one geographical region in the subpopulation. In theory, the low use farms would have had either no or a low level of selection pressure on the bacteria present leading to a lower probability of resistant bacteria being favoured in the farm environment. In contrast, the high use farms could have had such a broad use of antimicrobials such that even ESBL-producing *E. coli* were not able to thrive. Nevertheless, we do not believe either of these cases to be true, as we determined no statistically significant difference between the prevalence of ESBL-producing *E. coli* on high or low AMU farms in this study, and the overall AMU on Austrian dairy farms is known to be relatively low (even the maximum use of antimicrobials on the highest using farm was <8.10 DDD/cow/year (median 3.82 DDD/cow/year), compared to other countries [44,45,47,48,49]).

## 4. Materials and Methods

### 4.1. Study Population

The sample of farms included in this pilot study was taken from a larger nationwide study of 248 Austrian dairy farms, as described elsewhere [20,44]. Overall antimicrobial use (AMU) was analysed as part of the previous study [44] and a subset of 50 farms in the federal state of Styria were chosen to participate in this pilot study based on their total AMU over a one-year period. These farms were selected to include the 25 highest users of all AMU and the 25 lowest users in the federal state who were participating in the larger study. The analysis of AMU was based on the Defined Daily Dose metric (DDDvet) as assigned for each antimicrobial active ingredient by the European Medicines Agency [21].

Farms were selected solely on their AMU calculated in DDDvet per cow per year in the previous year, no adjustments were made for housing system, production system (organic or conventional), herd size, or any other farm-related factors.

Bacteriological culture results from milk samples taken from these farms over the one-year study period were available, as described in detail for all 248 farms elsewhere [50]. The most common mastitis pathogens in the larger study population were *Staphylococcus* spp. (40.1%), *Streptococcus* spp. (24.1%), and *Enterobacteriaceae* (13.3%) [50]. A total of 3020 quarter samples from 647 cows on 166 farms were analysed as part of the wider study and it was determined that multiresistant (MDR, i.e., resistant to at least 3 antimicrobials from different classes [51]) strains were most common among *Enterobacteriaceae* spp., with 14.3% (19/133) of isolates, followed by *Staphylococcus* spp. with 5.5% (22/402) MDR isolates [50].

### 4.2. Background Information on Udder Health and Herd Management

Detailed information on the farms included in this pilot study is available in the form of questionnaire responses (see Supplementary Materials), as previously described with respect to mastitis risk factors and overall farm management [20].

### 4.3. Total Antimicrobial Use in Defined Daily Dose (DDDvet/Cow/Year)

Total AMU was calculated from veterinary treatment records covering 1 October 2015 to 30 September 2016, as detailed elsewhere [44,52]. A total of 11 veterinary practices provided data for this pilot study of 50 farms. Briefly, the number of DDDvet were calculated for each antimicrobial substance by dividing the total amount of active substance in milligrams by the European Medicines Agency’s predefined DDDvet values [21].

Intramammary treatments were calculated slightly differently, as the European Medicines Agency classes each udder tube as 1 DDDvet, regardless of milligrams of active substance or standardised cow live weight (for details, see [21,44]).

DDDvet values have not been assigned by the EMA for dry cow therapy (DCT) intramammary tubes, only Defined Course Doses (DCDvet). For this reason, DCT was excluded from the majority of the AMU analyses included here. However, as dry cow therapy is an important part of the antimicrobial use statistics of dairy farms, a comparison of the corrected DCDvet/cow/year was carried out between those farms testing positive for the presence of ESBL-producing *E. coli* and those testing negative. The DCD/cow/year value was corrected by the replacement rate and calving interval for each of the farms included here, as described elsewhere [53].

No antimicrobial treatments were excluded in the present study (with the exception of unquantifiable sprays), regardless of diagnosis.

### 4.4. Farm Sampling and Bacteriological Culture

Each farm was sampled by one of two authors (CLF and WO). Personal protective equipment (PPE) in the form of disposable overalls, gloves, and boot covers were used to ensure that each site was not contaminated. As many Austrian dairy farms keep dual-purpose Simmental cows (Austrian *Fleckvieh*) for milk production, they frequently rear a number of female replacement heifers, as well as some male animals for beef production. For this reason, cows, calves, and youngstock are often found in the same building or on the same farm.

Each farm was visited in July 2017 and the following samples were collected:

- 2 pairs of boot swabs either from the alleyways of freestalls where lactating cows were housed or the slurry passage immediately behind cows in tie-stalls
- 1–2 pooled faecal samples from calf pens or freshly voided faeces if calves defaecated while researchers were present (1–5 calves per sample; 2 pooled samples were taken from farms with >50 head of cattle)
- 1–2 pooled faecal samples from youngstock (>6 months) pens (if youngstock were present on the farm, 1–5 head per sample; 2 pooled samples were taken from farms with >50 head of cattle)

Additionally, one dust sample was collected on dry gauze from 3 to 5 faeces-free areas of the main cowshed (for the isolation of MRSA).

Samples were immediately stored in an insulated cool box until they could be refrigerated. Faecal samples were transported to the laboratory of the Styrian Provincial Government Veterinary Authorities in Graz. Approximately 2 g faeces from each sample were pre-enriched in 200 mL buffered peptone water (BPW) at 37 °C for 18–24 h and then subsequent selective plated on ChromID-ESBL agar (bioMerieux, Marcy-L’Etoile, France). Plates were incubated at 37 °C for 24–28 h. Broth microdilution of the presumptive ESBL-producing *E. coli* was performed using the Sensititre™ EU Surveillance ESBL (EUVSEC2) plate (Thermo Fisher Scientific, Waltham, MA, USA) according to the manufacturer’s instructions (briefly, each well was filled with 50 µL inoculum, the plate was then sealed and incubated at 34–36 °C for 18 h in a non-CO2 incubator). The Sensititre™ EU Surveillance ESBL (EUVSEC2) plate includes wells containing cefotaxime and ceftazidime with or without clavulanic acid, as well as cefoxitin, ertapenem, imipenem, meropenem, cefipime, temocillin, and positive control wells. An ESBL phenotype was categorised according to the EFSA/ECDC standard EU surveillance method, namely a minimal inhibitory concentration (MIC) >1 mg/L for cefotaxime and/or ceftazidime, a positive synergy test for these antimicrobials with clavulanic acid, an MIC ≤8 mg/L for cefoxitin, and an MIC ≤ 0.12 mg/L for meropenem [54].

Dust samples for MRSA isolation were transported to the National Reference Laboratory for Antimicrobial Resistance at the Institute for Medical Microbiology & Hygiene, Graz (part of the Austrian Agency for Health and Food Safety (AGES)). Swabs were inoculated in 100 mL Mueller–Hinton broth supplemented with 6.5% sodium chloride (NaCl) and incubated for 16–20 h at 37 °C. A 10 μL loopful of pre-enrichment broth was spread on chromID MRSA agar (bioMerieux, Marcy-L’Etoile, France) and incubated for 24–48 h at 37 °C. In addition, 1 mL of pre-enrichment broth was added to 9 mL tryptone soya broth (TSB) containing 3.5 mg/L cefoxitin and 75 mg/L aztreonam and incubated for another 16–20 h at 37 °C. A total of 10 µL of the selective enrichment broth were spread on chromID MRSA agar (bioMerieux) and incubated for 24–48 h at 37 °C. Presumptive MRSA colonies were confirmed by Time of Flight Mass Spectrometry (MALDI-TOF MS) and subsequent PCR targeting mecA and mecC according to methods described elsewhere [55,56], with minor deviations (for details, see the Supplementary Materials).

### 4.5. Statistical Analysis

Statistical analyses were carried out using the SPSS (IBM, Armonk, NY, USA) statistical software and Microsoft Excel (Microsoft Corporation, WA, USA). A non-parametric test of independent samples, the Mann–Whitney U-test, was done using the total DDDvet/cow/year for each of the 50 farms and whether at least one sample from the farm tested positive for the presence of ESBL-producing *E. coli*. This test was then repeated using the DDDvet/cow/year for third and fourth generation cephalosporins for each farm. Furthermore, using 2 × 2 contingency tables, the Chi-squared test was carried out on group sizes larger than five, while contingency tables where one or more category was smaller than five were assessed using Fisher’s *t*-test. Statistical significance was set at *p* < 0.05 in all cases.

## 5. Conclusions

The level of antimicrobial resistance on the study farms was assessed by means of environmental samples collected from 50 dairy farms in the Austrian federal state of Styria chosen for either their high or low levels of overall AMU over a one-year period. The most frequent indication for antimicrobial use was the treatment of udder disease. No statistically significant relationships between high AMU in DDDvet/cow/year and the presence of these AMR bacteria were determined. While the low prevalence of ESBL-producing *E. coli* in cattle determined here correspond with national AMR monitoring of young calves, our findings may also have been due to the generally low level of antimicrobial use and subsequent lack of selection pressure for resistance genes on the study farms and, therefore, further investigation is needed with a larger sample size.

## Figures and Tables

**Table 1:** Demographics of study farms, total AMU in DDDvet/cow/year, and results of ESBL-producing *E. coli* screening.

	HIGH (N = 25)		LOW (N = 25)	
DDDvet/Cow/Year				
Range	2.47–8.04 3.82 4.35		0.01–0.63 0.31 0.29	
Median				
Mean				
	**Freestall (*n* = 17)**	**Tie-stall (*n* = 8)**	**Freestall (*n* = 16)**	**Tie-stall (*n* = 9)**
**Production system**				
Conventional	12	5	11	3
Organic	2	0	5	2
No answer given ^#^	3	3	0	4
Waste milk * routinely fed to calves				
Yes	12	3	9	3
No	2	2	7	2
No answer given ^#^	3	3	0	4
**ESBL-producing *E. coli* present on farm**				
Cowshed boot swabs	2	1	3	1
Calf samples	3	1	5	2
Youngstock samples	2	2	0	0
**Total number of farms with at least one positive ESBL-producing *E. coli* sample**	3	2	6	2
				
**Total number of farms where all three samples were ESBL-producing *E. coli* positive**	2	1	0	0

* Waste milk is defined here as non-saleable milk, usually containing antimicrobial residues, or within the milk withholding period. ^#^ Farmer did not complete farm management survey; therefore this information is not available for this farm.

**Table 2:** Antimicrobial treatments by disease indication, according to DDDvet/cow/year, and whether farms tested positive or negative for ESBL-producing *E. coli.*

	Proportion of Overall Antimicrobial Treatments (%) Based on Total DDDvet/Cow/Year	Proportion of Antimicrobial Treatments by Disease Indication	
		Non-HPCIA *	HPCIA *
ESBL-POSITIVE FARMS (N = 13)			
**Respiratory disease**	13.2%	54.8%	45.2%
**Musculoskeletal/Locomotory disease**	5.2%	32.5%	67.5%
**Udder disease (excluding DCT ^#^)**	52.2%	79.0%	21.0%
**Reproductive disorders**	20.4%	99.4%	0.6%
**Other diseases**	9.0%	47.8%	52.2%
ESBL-NEGATIVE FARMS (N = 37)			
**Respiratory disease**	12.4%	78.1%	21.9%
**Musculoskeletal/Locomotory disease**	8.4%	10.6%	89.4%
**Udder disease (excluding DCT ^#^)**	54.1%	37.4%	62.6%
**Reproductive disorders**	16.4%	95.2%	4.8%
**Other diseases**	8.6%	45.4%	54.6%

* HPCIA—highest priority critically important antimicrobials as defined by the World Health Organization [22]. For this study, HPCIA included third and fourth generation cephalosporins, macrolides, and fluoroquinolones. ^#^ DCT—dry cow therapy.

**Table 3:** 2 × 2 contingency table with respect to high and low AMU. Observed frequencies of ESBL-producing *E. coli* positive or negative farms (expected frequencies in brackets).

	High AMU Group ≥2.47 DDDvet/Cow/Year	Low AMU Group ≤0.63 DDDvet/Cow/Year	Total
ESBL-producing *E. coli*—positive	5 (6.5)	8 (6.5)	13
ESBL-producing *E. coli*—negative	20 (18.5)	17 (18.5)	37
Total	25	25	50

Chi-squared test statistic 0.93, *p* = 0.33.

**Table 4:** 2 × 2 contingency table with respect to numbers of dairy cows. Observed frequencies of ESBL-producing *E. coli* positive or negative farms (expected frequencies in brackets).

	≤20 Dairy Cows	>20 Dairy Cows	Total
ESBL-producing *E. coli*—positive	5 (6.2)	8 (6.8)	13
ESBL-producing *E. coli*—negative	19 (17.8)	18 (19.2)	37
Total	24	26	50

Chi-squared test statistic 0.64, *p* = 0.42.

**Table 5:** 2 × 2 contingency table with respect to whether calves were fed waste milk containing antimicrobial residues on the farm. Observed frequencies of ESBL-producing *E. coli* positive (at least one positive sample) or negative farms.

	Waste Milk Fed to Calves	Waste Milk NOT Fed to Calves	Total
ESBL-producing *E. coli*—positive	5	4	9
ESBL-producing *E. coli*—negative	22	9	31
Total	27	13	40 *

Fisher’s exact test, *p* = 0.44. * Details on which farms fed waste milk were obtained via a questionnaire, which was completed by 40/50 of the farmers included in this study.

## Data Availability

The authors do not own the antimicrobial use data analysed here. These data were collected from the veterinarians who were treating the animals included in the study. Under the data privacy agreement signed by the farmers and veterinarians, these data are not available to be published.

## Supplementary Materials

The following supporting information can be downloaded at: https://www.mdpi.com/article/10.3390/antibiotics11020124/s1, S1: English summary of the farm management questionnaire; S2: Description of PCR methods.
