# Supplementary material for: Analysis of Antimicrobial Use and the Presence of Antimicrobial-Resistant Bacteria on Austrian Dairy Farms—A Pilot Study
Source: Antibiotics (Basel). 2022 Jan 18;11(2):124. doi: 10.3390/antibiotics11020124 (PMC8868072; doi:10.3390/antibiotics11020124)
Supplement: Supplementary file 1 [file antibiotics-11-00124-s001.zip › antibiotics-1545451-Supplementary-update.pdf]

Analysis of antimicrobial use and the presence of antimicrobial-resistant bacteria on Austrian dairy farms – a pilot study

Supplementary Material S1

Three online surveys were completed by farmers, herd veterinarians, and national milk recorders. The complete surveys in German are available from the authors upon request. Here, a summary of survey aspects relevant to udder health is provided in English.

(This translation has previously been published in the Supplementary Materials of "Firth, C.L. et al. Relationship between the probability of veterinary-diagnosed bovine mastitis occurring and farm management risk factors on small dairy farms in Austria. J. Dairy Sci. 2019, 102, 4452–4463, doi:10.3168/JDS.2018-15657.)

Question	Source	Possible questionnaire responses
Age of dairy cow	Herd data	
Altitude of farm	Herd data	
Lactation number	Herd data	
Herd size	Herd data	
Main production areas	Herd data	Various alpine and lowland regions
Are farms managed full-time or part-time?	Farmers' questionnaire	Full-time<br>Part-time
How often is a bacteriological culture of mastitic milk carried out?	Farmers' questionnaire	Not regularly<br>Regularly
California mastitis test (CMT) is carried out?	Farmers' questionnaire	Never<br>Symptomatic animals<br>All animals
Type of production system?	Farmers' questionnaire	Conventional<br>Organic
Is a bacteriological culture of milk carried out before drying off?	Farmers' questionnaire	Never<br>Symptomatic animals<br>Regularly
How are cows routinely dried off?	Farmers' questionnaire	Abruptly<br>Gradually
Are cows routinely dried off with antibiotics?	Farmers' questionnaire	Yes, all animals<br>Symptomatic animals or no
Are teat sealants used at drying off?	Farmers' questionnaire	Never<br>Occasionally<br>Regularly
Are dry cows' udders regularly checked (includes visual check)?	Farmers' questionnaire	Never/rarely<br>Occasionally

(C) Firth et al, 2022

		Regularly
Separate calving box available?	Farmers' questionnaire	Yes<br>No
Housing system	Farmers' questionnaire	Tie-stall<br>Freestall
Is waste milk fed to calves?	Farmers' questionnaire	Yes – all calves<br>Yes – only bull calves<br>No
Who milks the cows regularly?	Farmers' questionnaire	1 Person<br>More than one person<br>AMS
How often are the cubicles/lying areas cleaned?	Farmers' questionnaire	Twice a day<br>Once a day<br>Less frequently
How often is the bedding material replaced/freshened?	Farmers' questionnaire	Daily<br>Less often
Disinfection of cows' beds?	Farmers' questionnaire	Yes<br>No
Age of the farm buildings?	Farmers' questionnaire	<= 10 years<br>> 10 years
Is there a convalescence box available?	Farmers' questionnaire	Yes<br>No
Insect prevention on farm?	Farmers' questionnaire	Irregularly<br>Regularly
How often are the cows fed?	Farmers' questionnaire	Once a day<br>Twice a day<br>Three times a day<br>Permanent access to feed
Are cows fed immediately after milking?	Farmers' questionnaire	Yes<br>No
Is there enough space along the feed passage for all cows to eat simultaneously?	Farmers' questionnaire	Yes<br>No
Do cows have regular access to pasture?	Farmers' questionnaire	Yes<br>No
Do cows have access to an outdoor loafing area?	Farmers' questionnaire	Yes<br>No
Are homeopathic remedies used to treat udder disease?	Farmers' questionnaire	Yes<br>No
Energy-corrected milk (kg) (varies according to cow breed)	National milk performance recorder data	
Duration of milking (days)	National milk performance recorder data	
Is a disinfectant (e.g., iodine) used to clean the teats?	NMR questionnaire	Yes<br>No
Is there a milking order? (Are cows with udder disease milked last?)	NMR questionnaire	Yes<br>No<br>Separate cluster
Does the milking machine have an automatic shut off?	NMR questionnaire	Yes<br>No

(C) Firth et al, 2022

Are milkers carrying out other jobs (e.g. feeding calves) while milking?	NMR questionnaire	Yes<br>No<br>AMS
Do the milkers wash/clean their hands before milking?	NMR questionnaire	Yes<br>No<br>AMS
Are gloves worn when milking?	NMR questionnaire	Yes<br>No<br>AMS
Teat dipping is carried out with…	NMR questionnaire	Non-return dip cup<br>Standard dip cup<br>Spray<br>Not done<br>AMS
Are the clusters disinfected between cows?	NMR questionnaire	Yes, between all cows<br>Yes, only between symptomatic cows<br>No
Type of milking system?	NMR questionnaire	Milking parlor (+ any other type)<br>Vacuum line (+ any other type)<br>Bucket<br>AMS
How frequently are the liners in the clusters changed?	NMR questionnaire	2-4 times a year<br>Once a year<br>Less frequently than once a year
Cleanliness of the milking equipment/parlor?	NMR questionnaire	Clean<br>Moderately to severely dirty
Is straw contained in the bedding material used for lactating cows?	NMR questionnaire	Yes<br>No
Is sawdust contained in the bedding material used for lactating cows?	NMR questionnaire	Yes<br>No
Is forestripping done?	NMR questionnaire	Yes<br>No
Udder and teat cleaning is carried out…	NMR questionnaire	Wet (e.g. with water)<br>Damp (e.g. with foam)<br>Dry (e.g. with paper)
Udder and teat cleaning is carried out using single use methods (e.g. paper, disposable cloths)?	NMR questionnaire	Yes<br>No
Hygiene score for udders prior to milking (estimated for the entire herd, based on a photographic scale)	Herd vet questionnaire	Clean-slightly soiled<br>Moderately – severely soiled
General farm hygiene – alleys	Herd vet questionnaire	Clean<br>Moderately soiled<br>Severely soiled
General condition of farm buildings where dairy cows are housed	Herd vet questionnaire	Good condition<br>Moderate<br>Poor condition
General assessment of overall farm hygiene	Herd vet questionnaire	Good<br>Not good (includes moderate and poor)

(C) Firth et al, 2022

Dairy cow breed	Cattle breeders association	Austrian Fleckvieh (Simmental)<br>Braunvieh (Brown Swiss)<br>Holstein-Friesian

Supplementary Materials S2

PCR Methods

Primers used for amplification of mecA were mecA F 5´TGGCTCAGGTACTGCTATCCAC and mecA R 5´AGTTCTGCAGTACCGGATTTG C.
DNA amplification was carried out for mecA as follows: 95°C for 1 min followed by 30 cycles with 95°C for 30 sec, 55°C for 30 sec, 72°C for 30 sec; and finally 72°C for 4 min.

Primers used for amplification of mecC were mecC F 5´GCTCCTAATGCTAATGCA and mecC R 5´TAAGCAATAATGACTACC.
DNA amplification was carried out for mecC as follows: 95°C for 10 min followed by 30 cycles with 95°C for 20 sec, 52°C for 20 sec, 72°C for 2 min; and finally 72°C for 10 min.

et al, 2022

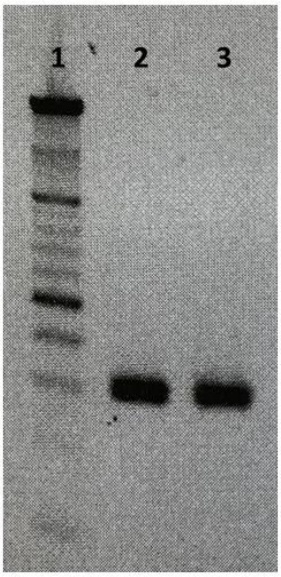

Fig. Agarose gel electrophoresis of amplified 304-bp *mecC* DNA fragment. Lanes: 1, 100-bp molecular weight marker (dark band was at 500 bp); 2, control strain *S. aureus* LGA251; 3, *S. aureus* strain 17/048.
